# A Protocol for Transcriptome-Wide Inference of RNA Metabolic Rates in Mouse Embryonic Stem Cells

**DOI:** 10.3389/fcell.2020.00097

**Published:** 2020-02-27

**Authors:** Adriano Biasini, Ana Claudia Marques

**Affiliations:** Department of Computational Biology, University of Lausanne, Lausanne, Switzerland

**Keywords:** RNA metabolic labeling, RNA metabolic rates, degradation rate, transcription rate, processing rate, MESC, 4sU, 4sU-RNA labeling

## Abstract

The relative ease of mouse Embryonic Stem Cells (mESCs) culture and the potential of these cells to differentiate into any of the three primary germ layers: ectoderm, endoderm and mesoderm (pluripotency), makes them an ideal and frequently used *ex vivo* system to dissect how gene expression changes impact cell state and differentiation. These efforts are further supported by the large number of constitutive and inducible mESC mutants established with the aim of assessing the contributions of different pathways and genes to cell homeostasis and gene regulation. Gene product abundance is controlled by the modulation of the rates of RNA synthesis, processing, and degradation. The ability to determine the relative contribution of these different RNA metabolic rates to gene expression control using standard RNA-sequencing approaches, which only capture steady state abundance of transcripts, is limited. In contrast, metabolic labeling of RNA with 4-thiouridine (4sU) coupled with RNA-sequencing, allows simultaneous and reproducible inference of transcriptome wide synthesis, processing, and degradation rates. Here we describe, a detailed protocol for 4sU metabolic labeling in mESCs that requires short 4sU labeling times at low concentration and minimally impacts cellular homeostasis. This approach presents a versatile method for in-depth characterization of the gene regulatory strategies governing gene steady state abundance in mESC.

## Introduction

Gene expression control is central to ensure appropriate responses to intrinsic and extrinsic cellular stimuli and cellular homeostasis. The steady state abundance of RNA transcripts is controlled by the rates at which the gene is transcribed (transcription), processed (processing), and degraded (degradation). Understanding how these three RNA metabolic rates change in response to different cellular cues, is paramount for in-depth characterization of how gene expression regulation, in health and disease, contributes to the maintenance or changes in cell state during development and in adulthood.

Different methods allow measurement of the individual contribution of each of these RNA metabolic rates to steady state abundance. Some of the most widely used techniques are based on the use of transcription inhibition or transcription synchronization drugs, such as 1-β-D-ribofuranoside (DRB), α-Amanitin (α-Ama) or Actinomycin-D (Act-D) (Alpert et al., 2017; Wada and Becskei, 2017). While these approaches were initially used to assess transcript-specific rates, the advent of next generation sequencing technologies now allow their transcriptome-wide implementation. The main limitation of these approaches is that transcription-inhibition induces cellular stress and can lead to a number of pleotropic effects. For example, since transcription and degradation rates have been suggested to be inherently linked (Haimovich et al., 2013), inhibiting transcription may thereby impact degradation rates and influence transcript half-live measurements. Transcriptional inhibitors have also been shown to lead to stabilization of proteins involved in gene expression control, such as p53, suggesting that secondary changes are also likely to impact the accuracy of the measurements (reviewed in Bensaude, 2011). Furthermore, it has also been suggested that decay rates of short- or long-lived transcripts cannot be accurately measured using approaches based on transcription blockage (Clark et al., 2012).

**FIGURE 1:**
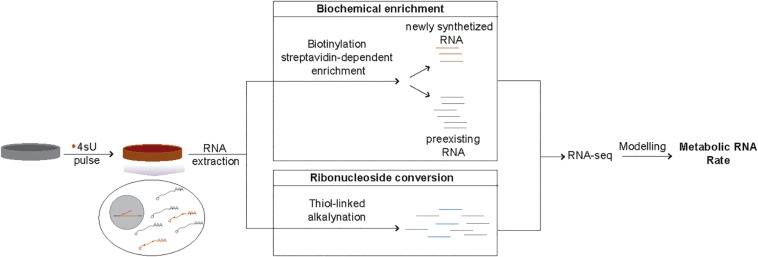
Schematic representation of 4sU metabolic labeling-based inference of RNA metabolic rates. Isolation of newly transcribed RNAs from the total RNA of cells treated with 4sU (orange) can be achieved in two ways. Biochemical enrichment relies on the biotinylation of 4sU incorporated newly transcribed RNAs, followed by streptavidin-dependent separation of the newly synthetized (4sU-incorporated, orange lines) transcripts from the preexisting RNA fraction (gray lines). Ribonucleoside conversion, on the other hand, is based on chemical induced conversion of 4sU into a base that is likely to be read as cytosine (blue lines) and can be distinguished from the preexisting RNA (gray lines) based on the presence of T-C changes. RNA-sequencing-based estimates of the relative levels of newly synthetized transcripts coupled with appropriate computational modeling approaches allows the inference of metabolic RNA rates.

Methods based on RNA metabolic labeling using modified nucleotides, such as 5′-Bromouridine (BrU) or 4-thiouridine(4sU), overcome these limitations (Baptista and Dolken, 2018). Specifically, this class of methods allows estimation of RNA metabolic rates with minimal impact on cellular homeostasis for most transcript classes (de Pretis et al., 2015). Metabolic labeling is based on the incorporation of modified ribonucleosides into nascent RNA transcripts during cellular proliferation. Compared to BrU, 4sU is more rapidly incorporated (reviewed in Tani and Akimitsu, 2012), and is the most widely used modified ribonucleoside. Isolation of transcripts synthesized during the incubation with 4sU from preexisting transcripts can be achieved by thiol-specific biotinylation of 4sU labeled RNA followed by streptavidin-dependent enrichment. Alternatively, chemical conversion of modified ribonucleosides can also be used to distinguish newly synthesized and preexisting transcripts (Herzog et al., 2017). Finally, transcriptome-wide RNA metabolic rates can be inferred by quantification of transcript levels in both RNA fractions coupled with computational modeling (Figure 1). Different mathematical modeling approaches have been developed to infer RNA metabolic rates from these types of data (Rabani et al., 2014; de Pretis et al., 2015; Lugowski et al., 2018; Neumann et al., 2019).

In its simplest form, RNA metabolic labeling experimental design relies on a single labeling time, similar to what is described here. Single labeling reduces experimental cost and complexity, likely at the expense of the accuracy of the degradation rate estimates (Wada and Becskei, 2017). Alternatively, the dynamics of nucleotide incorporation can also be explored to increase rate inference accuracy using approach-to-equilibrium or pulse-chase designs (Duffy et al., 2019).

RNA metabolic labeling using 4sU (Dolken et al., 2008), has been applied for inference of RNA metabolic rates of diverse transcript classes from highly stable microRNAs (Marzi et al., 2016) to rapidly decaying transcripts, such as long non-coding RNAs, in a wide range of cell lines (Marzi et al., 2016; Mukherjee et al., 2017; Freimer et al., 2018). Despite the widespread implementation of this method, data for transcriptome-wide metabolic rates in mESC – one of the most widely used model systems for the study of cell state homeostasis and cell state transitions – is limited (Freimer et al., 2018). Given the availability of numerous constitutive and inducible mESC mutants, analysis of RNA metabolic labeling in these cells can provide a better understanding how different genes and pathways modulate gene expression. Here we present a detailed protocol based on a short pulse with low concentrations of 4sU for RNA metabolic labeling in mESCs. This approach allows non-invasive (Supplementary Note Figures 1A–C) quantification of metabolic rates for most transcript classes including very short-lived RNAs. Due to its negligible impact on cell state and viability, this protocol can be effectively applied to wild-type and mutant mESC lines. In addition, we provide details on quality controls, that we adapted for mESC based on previous work (Radle et al., 2013), and that allow the user to assess the labeling and RNA quality throughout the experiment.

## Materials and Reagents

(A) Cell culture.
  (1) 10 cm tissue treated cell culture plate.
  (2) Knockout DMEM mESC growth medium (Thermo Fisher, 10829018):
    (i) 15% (v/v)Fetal Bovine Serum (Thermo Fisher, 16000044).
    (ii) 50 U/ml of Penicillin and 50 μg/ml of Streptomycin (Thermo Fisher, 15070063).
    (iii) 1 U/μl Recombinant Mouse LIF Protein (MERCK, ESG1107).
    (iv) 0.06 mM β-Mercaptoethanol (Thermo Fisher, 31350010).
    (v) 1% (v/v) 100 X Non-Essential Amino Acids (Thermo Fisher, 11140050).
  (3) Sterile 0.2% (m/v) Gelatin in H_2_O.

(B) 4-Thouridine labeling and total RNA extraction.
  (1) 4-thiouridine (Sigma, T4509) dissolved in DEPC-treated H_2_O (DEPC-H_2_O).
    *NOTE_1_: Keep at −20*°*C, protected from light (4sU is light-sensitive). Discard remaining 4sU solution after thawing.*
  (2) PBS.
  (3) Trizol reagent (Thermo Fisher, 15596026).
  (4) Chloroform (Sigma, C2432).
  (5) 15 ml phase lock-tubes (Qiagen, 129065).
  (6) 15 ml Falcon Tubes.
  (7) 1.5 ml tube (RNase and DNase free).
  (8) 5M NaCl in DEPC-H_2_O.
  (9) Isopropanol.
  (10) DEPC- H_2_O.
  (11) 75% (v/v) Ethanol in DEPC- H_2_O.
  (12) 5 ml Serological Pipette.
  (13) Qiagen RNeasy kit (Qiagen, 74104).
  (14) RNAse Free DNAse Set (Qiagen, 79254).

(C) Dot-blot quality control.
  (1) Zeta-Probe Membrane (Biorad, 1620190).
  (2) Biotin-labeled DNA oligo.
  (3) DEPC- H_2_O.
  (4) 2 ml Phase Lock tubes (Qiagen, 129056).
  (5) EZ-link Iodoacetyl-PEG2-biotin (Thermo Fisher, 21334) dissolved to 1 mg/ml in DMF (Thermo Fisher, 20673).
  (6) Na_2_HPO_4_ (Applichem, A1046,1000).
  (7) 20% Sodium Dodecyl Sulfonate (SDS) (Applichem, A0675,0250).
  (8) Phenol:Chloroform:isoamyl alcohol (Sigma, P2069-100ML).
  (9) PBS.
  (10) Streptavidin-horseradish peroxidase (Strep-HRP, Thermo Fisher, 21130).
  (11) Blocking Buffer: (0.5M Na_2_HPO_4_, 7% SDS, pH 7.2 in DEPC-H_2_O).
  (12) Wash solution 1: 40 ml PBS + 10% (v/v) SDS.
  (13) Wash solution 2: 40 ml PBS + 1% (v/v) SDS.
  (14) Wash solution 3: 40 ml PBS + 0.1% (v/v) SDS.
  (15) Advansta WesternBright ECL (advansta, K-12045-D50).

(D) RNA biotinylation for isolation of newly transcribed RNA.
  (1) 1.5 ml Eppendorf tubes (RNAse and DNAse free).
  (2) 10 X Biotinylation Buffer (100 mM Tris-HCl pH 7.4, 10 mM EDTA in DEPC-H_2_O).
  (3) 1 mg/ml EZ-link HPDP-Biotin (Thermo Fisher, 21341) in dimethylformamide (DMF) (Sigma, D4551).
  (4) Phenol:Chloroform:isoamyl alcohol (Sigma, P2069-100ML).
  (5) 2 ml phase lock tubes (Qiagen, 129056).
  (6) 75% (v/v) Ethanol in DEPC-H_2_O.
  (7) DEPC-H_2_O.
  (8) 2.0 ml Eppendorf tubes (DNAse and RNAse free).

(E) Bead preparation.
  (1) Dynabeads MyOne T1 Streptavidin Beads (Thermo, 65601).
  (2) 2X Bind and Wash (B&W) Buffer (10 mM Tris-HCl pH7.5, 1 mM EDTA, 2M NaCl in DPEC-H_2_O).
  (3) Solution A (0.1M NaOH, 0.05M NaCl in DEPC-H_2_O).
  (4) Solution B (0.1M NaCl in DEPC-H_2_O).

(F) Separation of newly transcribed RNA.
  (1) 1X B&W Buffer (5 mM Tris-HCl pH 7.5, 0.5 mM EDTA, 1M NaCl in DEPC-H_2_O).
  (2) RNeasy Mini kit (Qiagen, 74104).
  (3) RNase-Free DNase Set (Qiagen, 79254).
  (4) Isopropanol.
  (5) 5M NaCl in DEPC- H_2_O.
  (6) Isopropanol.
  (7) 75% Ethanol in DEPC-H_2_O.
  (8) RNase-free DEPC-H_2_O.
  (9) 70% Ethanol in DEPC-H_2_O.
  (10) 100 mM Dithiothreitol (DTT) (Thermo Fisher, R0861) in DEPC-H_2_O.

(G) RT-qPCR analysis.
  (1) SuperScript IV First-Strand Synthesis System (Thermo Fisher, 18091200).
  (2) FastStart Essential DNA Green Master (Roche, 06402712001).
  (3) Gene specific primers (sequences can be found in Supplementary Table 1).
  (4) DEPC-H_2_O

**Equipment.**

(A) Cell culture Hood (Thermo Fisher, Herasafe KS12).
(B) Chemical Hood (Waldner, Bench-mounted fume hood).
(C) Cell culture incubator at 37°C with 5.0% CO_2_ (Thermo Fisher, Direct Heat CO_2_ Incubator).
(D) Centrifuge with temperature control (Beckman Coulter, Avanti J-26 XP).
(E) Standard tabletop centrifuge with temperature control (Eppendorf, Centrifuge 5415 R).
(F) Rotating Vertical Mixer (Stuart, Rotator SB3).
(G) DynaMag-2 Magnet Magnetic Rack (Thermo Fisher, 12321 D).
(H) Quantitative PCR Machine (Roche, Light Cycler 96).
(I) See-saw rocker (Stuart, See-saw rocker SSL4).
(J) Chemiluminescence detection apparatus (Vilber Lourmat, FUSION Solo 6S Imaging System).
(K) Thermocycler (Biometra Thermocycler ThermoBlock).
(L) Fluorescent Activated Cell Sorter (Synergy MX flow Cytometer Fluorescent activated Cell Sorter).
(M) Nanodrop (ND-1000 Spectrophotometer).

**Methods.**

(A) Before You start:
  (1) Centrifuge all 2 ml phase lock tubes and 15 ml phase lock tubes at 12,000 × *g* for 30 s and 1,500 × *g* for 60 s respectively.
  (2) Cool ultracentrifuges and tabletop centrifuges to 4°C.

(B) 4-Thouridine labeling and total RNA extraction.
  (1) The day before labeling, seed mESCs in two gelatin-coated 10 cm plates (12 ml of growth medium). Cells in one plate will be labeled with 4sU whereas cells in the other plate will be untreated and will serve as negative control.
    NOTE_2_: mESCs should be 70-80% confluent at the time of labeling.
  (2) Transfer 7 ml of medium from one of the overnight mESC culture (4sU-treated mESC) to a 15 ml falcon tube, add 4sU to a final concentration of 200 μM and mix thoroughly by pipetting up and down.
    *NOTE_3_: Negative control plate (containing untreated cells) should be kept in a humidified cell culture incubator at 37*°*C with 5% CO_2_ until RNA is harvested (step 5). From that point onwards RNA from 4sU treated and untreated cells should be handled in parallel.*
  (3) Remove the remaining 5 ml of growth medium in the plate and add 7 ml of 4sU supplemented growth medium.
    *NOTE_4_: When planning to use different final 4sU concentrations one should consider the following: (1) High 4sU concentrations can induce nucleolar stress responses and impact proliferation rates* (Burger et al., 2013; Radle et al., 2013)*; (2) Low 4sU concentrations may result in low recovery of newly transcribed RNA.*
  (4) Incubate cells in a humidified cell culture incubator at 37°C with 5% CO_2_, for the desired pulse duration.
    NOTE_5_: The “Representative results”, described below were obtained from a 15 min 4sU pulse.
  (5) Aspirate media and wash plates with 4 ml PBS.
  (6) Inside a chemical hood, add 5 ml of Trizol reagent to plates. Ensure Trizol covers the whole surface of the plate.
  (7) Incubate at room temperature for 5 min.
  (8) Transfer mESC cell lysate in Trizol from plates to clean 15 ml falcon tubes.
    NOTE_6_: Lysate- containing Trizol can be stored at 4°C for up to 12 h.
  (9) Add 1 ml of chloroform to lysate-containing Trizol and mix vigorously by pipetting.
  (10) Transfer mixture to the prespun 15 ml phase lock tube.
  (11) Incubate, at room temperature, until clear separation between organic and inorganic phase is obtained (minimum 3 min).
  (12) Centrifuge for 5 min at 4°C at 1500 × *g*.
  (13) Carefully transfer the upper phase (contains RNA) to clean 15 ml falcon tube.
  (14) Add equal volume of Isopropanol (∼3.0–3.5 ml) and mix vigorously by pipetting.
  (15) Incubate mixture at room temperature for 10 min.
  (16) Centrifuge for 15 min at 4°C at 11000 × *g*.
    NOTE_7_: RNA pellet should be visible at the end of this centrifugation step.
  (17) Remove supernatant and wash RNA pellet with 3.5 ml of 75% Ethanol in DEPC-H_2_O.
  (18) Centrifuge for 5 min at 4°C at 7500 × *g*.
  (19) Remove supernatant carefully to avoid dislodging RNA pellet.
  (20) Resuspend RNA pellet in 100 μl of RNAse free DEPC-H_2_O and transfer to fresh 1.5 ml tube.
  (21) Remove genomic DNA. To this end, we use on-column DNAse I treatment (RNeasy Mini kit, Qiagen) according to manufacturer’s instructions.
    NOTE_8_: On-column DNase digestion using the RNeasy Mini kit may result in significant loss of small RNA species. If enriching for small RNA species we suggest using an alternative approach [for example TURBO^TM^ DNase (Thermo Fisher, AM2238)].
  (22) Adjust total RNA volume to 100 μl and quantify RNA concentration.
    NOTE_9_: Proceed directly to RNA Biotinylation or store RNA at −80°C.
    (C) Optional dot-blot for assessment of 4sU incorporation.
    (1) Assemble in a 1.5 ml RNAse/DNAse free tube, 300 μl biotinylation reaction by adding in the following order:
    (i) 30 μg of total RNA in 210 μl DEPC-H_2_O.
    (ii) 30 μl of 10X Biotinylation Buffer.
    (iii) 60 μl of EZ-link iodoacetyl-Biotin dissolved to 1 mg/ml in DMF.
    (2) Mix vigorously, by pipetting, until solution is homogenous.
    NOTE_10_: Ensure to mix immediately after addition of EZ-link iodoacetyl-Biotin to avoid precipitation.
  (3) Incubate the mixture on Rotating Vertical Mixer for 2 h at room temperature.
    *NOTE_11_: During incubation, precool a tabletop centrifuge to 4°C, centrifuge 2* × *2 ml phase lock tubes at 12000 × g for 30 s for each sample being tested and prepare blocking solution and wash solutions 1–3.*
  (4) Add 300 μl of phenol:chloroform:isoamyl alcohol to biotinylation reaction and mix vigorously by pipetting.
  (5) Add biotinylation reaction and phenol:chloroform:isoamyl mixture to prespun phase lock tubes and allow phases to separate at room temperature (minimum 3 min).
  (6) Centrifuge for 5 min at 4°C at 12000 × *g*.
  (7) Transfer the organic phase (∼280 μl) into a clean 1.5 ml tube.
  (8) Add equal volume of phenol:chloroform:isoamyl alcohol, mix by pipetting, transfer to phase lock tube and repeat steps 6–7 once.
  (9) Transfer organic phase (generally 275 μl) to clean 1.5 ml tube, add equal volume of isopropanol and 1/10 volume of 5M NaCl. Mix well by pipetting.
  (10) Centrifuge for 45 min at 4°C at 16000 × *g*. RNA pellet should be visible following this centrifugation step.
  (11) Carefully remove supernatant and resuspend RNA pellet in 300 μl 75% Ethanol in DEPC-H_2_O.
  (12) Spin for 10 min at 4°C at 13000 × *g*.
  (13) Carefully remove supernatant and resuspend pellet in 20 μl of DEPC-treated H_2_O.
  (14) Quantify RNA concentration, and maintain on ice until use.
  (15) Incubate Zeta-membane in 5-10 ml DEPC-H_2_O on a see-saw rocker for 10 min.
    NOTE_12_: Membrane should be covered by DEPC-H_2_O.
  (16) Take Zeta-membrane and remove excess liquid by dabbing both sides gently with clean paper towels.
  (17) Allow membrane to air dry for 5–10 min.
  (18) Prepare, for each RNA sample (including 4sU-untreated control), 10 μl of 1000 ng/ul dilution in DEPC-H_2_O.
  (19) Prepare four 1:2 serial dilutions (500, 250, 125, 62.5 ng/μl RNA) in 3 μl for each of the RNA samples, in DEPC-H_2_O.
  (20) Place Zeta-membrane on top of clean glass surface and apply 2 μl of each dilution of RNA to the zeta membrane. Additionally, add 2 μl of 100 ng/μl Biotinylated Oligo as positive control for Strep-HRP activity.
    NOTE_13_: To ensure proper spacing between blotted samples, we suggest pipetting the RNA onto the zeta membrane through the holes of an empty pipette tip box.
  (21) Air dry membrane for 7 min.
  (22) Incubate the membrane for 30 min in 30 ml of freshly prepared blocking buffer (0.5M NaH_2_PO_4_, 7% SDS, pH 7.2, in DEPC-H_2_O) on a see-saw rocker.
  (23) Remove blocking solution and incubate the membrane with 10 ml of freshly prepared 1:1000 streptavidin-horseradish peroxidase solution for 15 min (5 ml PBS + 5 ml 20%SDS + 10 μl of streptavidin horseradish peroxidase).
    NOTE_14_: Streptavidin horseradish-peroxidase should be thawed on ice and added just before use.
  (24) Wash membrane twice with Wash solution 1 for 10 min.
  (25) Wash membrane twice with Wash solution 2 for 10 min.
  (26) Wash membrane twice with Wash solution 3 for 10 min.
  (27) Proceed with chemiluminescent detection using WesternBright ECL solutions according to manufacturer’s instructions.

(D) RNA biotinylation.
  (1) Thaw RNA (from section B step 22), on ice.
  (2) Assemble in a 2.0 ml RNAse/DNAse free tube, 1.0 ml biotinylation reaction by adding in the following order:
    (i) 100 ug of RNA in 700 μl of DEPC-H_2_O.
    (ii) 100 μl of 10X Biotinylation Buffer.
    (iii) 200 μl of EZ-link Biotin HPDP dissolved to 1 mg/ml in DMF.
    NOTE_15_: Ensure to mix immediately after addition of EZ-link HPDP-Biotin to avoid precipitation.
    *NOTE_16_: To biotinylate different amounts of 4sU labeled RNA scale reagents proportionally. We do not recommend labeling less than 80 ug of RNA as in our hands and for short pulse durations this leads to* < *150 ng newly synthetized RNA.*
  (3) Mix vigorously, by pipetting, until solution is homogenous.
  (4) Incubate the mixture on Rotating Vertical Mixer for 2 h at room temperature.
  (5) Add equal volume (in this case 1.0 ml) of phenol:chloroform:isoamyl alcohol to the biotinylation reaction and mix vigorously by pipetting.
  (6) Add biotinylation reaction and phenol:chloroform:isoamyl mixture to prespun 2.0 ml phase lock tubes and allow phases to separate at room temperature (minimum 3 min).
    *NOTE_17_: We recommend using 2.0 ml phase lock tubes as in our experience using 15 ml phase lock tubes leads to considerable loss of material. As the maximum sample volume of 2 ml phase lock tubes is 750* μ*l, the mix obtained at the end of step 5 (2.0 ml total Volume) should be divided into 3 separate 2.0 ml phase lock tubes and processed separately until precipitation (step 9).*
  (7) Centrifuge for 5 min at 4°C at 12000 × *g*.
  (8) Repeat steps 5–7 to ensure complete removal of unreacted biotin that could otherwise interfere with binding of biotinylated RNA to streptavidin.
  (9) Transfer upper phase containing RNA into clean 2.0 ml tube and mix with 1/10 volume of 5M NaCl and an equal volume (∼850 μl) of Isopropanol.
  (10) Centrifuge for 45 min at 4°C at 16000 × *g*. RNA pellet should be visible following this centrifugation step.
    NOTE_18_: Bead preparation (section E) can be performed during this centrifugation step, assuming that on average 5–10% of RNA is lost during the biotinylation.
  (11) Remove supernatant and resuspend in equal volume (850ul) of 75% Ethanol.
  (12) Centrifuge for 10 min at 4°C at 16000 × *g*.
  (13) Remove supernatant, taking care not to dislodge the RNA pellet.
  (14) Resuspend RNA in 100 μl of DEPC-H_2_O and transfer to a clean 1.5 ml tube.
  (15) Quantify RNA concentration and keep RNA on ice until separation of newly transcribed RNA from preexisting RNA step.

(E) Bead preparation.
  NOTE_19_: Beads preparation is based on manufacturer’s instructions. For more details please refer to the manual provided with MyOne Streptavidin T1 Dynabeads by Thermo Fisher.
  (1) Vortex MyOne Streptavidin T1 Dynabeads for 30 s to ensure complete beads resuspension.
  (2) Pipette 2 μl of MyOne Streptavidin T1 Dynabeads for each μg of precipitated biotinylated RNA into a 1.5 ml tube.
  (3) Add equal volume (minimum 1.0 ml) of 1X B&W Buffer to beads.
  (4) Incubate on Rotating Vertical Mixer for 1 min at room temperature.
  (5) Place tube containing resuspended beads on Dynamag Magnetic Rack and separate beads for 1 min.
  (6) Carefully remove supernatant and resuspend beads in equal volume of 1X B&W Buffer.
  (7) Repeat steps 3-6 for a total of four washes in 1X B&W Buffer.
  (8) Resuspend beads in 1.0 ml of Solution A and incubate on a Rotating Vertical Mixer for 2 min.
  (9) Place on Dynamag Magnetic rack and separate beads for 1 min.
  (10) Remove supernatant carefully.
  (11) Repeat steps 8–10 once.
  (12) Resuspend beads in 1.0 ml of Solution B and incubate on Rotating Vertical Mixer for 2 min.
  (13) Place on Dynamag Magnetic Rack and separate beads for 1 min.
  (14) Remove supernatant carefully.
  (15) Repeat steps 12–14 once to remove NaOH traces.
  (16) Resuspend beads in same volume of 2X B&W Buffer as the volume of beads initially taken from the vial.
    NOTE_20_: Beads concentration for optimal coupling with RNA can be optimized for specific applications and pulse durations.

(F) Separation of newly transcribed RNA from preexisting RNA.
  (1) Resuspend biotinylated RNA precipitated in section D steps 14–15 to a final concentration of 500 ng/μl in DEPC-H_2_O.
  (2) Mix previously washed Dynabeads with equal volume of precipitated biotinylated RNA in DEPC- H_2_O by pipetting.
  (3) Place on Rotating Vertical Mixer and mix for 15 min at room temperature.
    *NOTE_21_: Beads and RNA solution is viscous! If solution viscosity inhibits even mixing, it is critical to add same volume of 1X B*&*W to all samples being processed to uniformly reduce bead concentration and ensure even mixing.*
  (4) Place biotinylated RNA coated beads on Dynamag Magnetic Rack for 3 min to separate beads from solution.
  (5) Remove and discard supernatant.
  (6) Resuspend beads in 500 μl of 1X B&W Buffer and mix on Rotating Vertical Mixer for 1 min.
  (7) Place tube on Dynamag Magnetic rack for 1 min.
  (8) Repeat steps 6–7 for a total of three washes with 1X B&W Buffer. Discard supernatant.
  (9) Resuspend beads in 100 μl freshly prepared 100 mM DTT and incubate at room temperature for 1 min.
  (10) Add 350 μl of Qiagen RNeasy Mini Kit Buffer RLT to DTT bead suspension and mix thoroughly by pipetting.
    NOTE_22_:If enriching for small RNAs we suggest Trizol for RNA elution from beads and Qiagen miRNeasy Mini Kit (Qiagen, 217004) according to manufacturer’s instructions for RNA purification.
  (11) Incubate at room temperature for 5 min.
  (12) Place mixture containing beads on Dynamag Magnetic Rack and allow to separate for 2 min.
  (13) Transfer supernatant to a clean 1.5 ml tube.
  (14) Add equal Volume of 70% EtOH in DEPC-H_2_O (450 ul) to tube and mix well by pipetting.
  (15) Proceed with RNA purification using the Qiagen RNeasy Mini Kit according to the manufacturer’s instructions.
  (16) Elute with 25 μl of DEPC-H_2_O and quantify concentration.

(G) Optional reverse transcription quantitative PCR analysis of newly transcribed and total RNA ratio.
  (1) Reverse transcribe the same volume of total RNA and newly transcribed RNA from each sample using random hexamers.
    *NOTE_23_: In the “Representative results” section we report on data obtained from reverse transcription of 2* μ*l of RNA using the SuperScript IV cDNA synthesis kit according to the manufacturer’s instructions.*
  (2) Measure the expression of transcripts that vary across a range of reported stabilities (*Ccne2, Myc, Actin-*β, *Rplp0*, *GapdH*) in the newly transcribed and total RNA fractions by RT-qPCR.
    *NOTE_24_: In the “Representative results” section we report the results for qPCR reactions performed in* Roche Lightcycler 96^®^ *according to manufacturer’s instructions. qPCR reaction was assembled by adding 2* μ*l of cDNA diluted 1:4, 5* μ*l of FastStart Essential DNA Green Master, gene specific primers to final concentration of 0.5* μ*M and 2.5* μ*l of DEPC-H_2_O.*
  (3) To calculate the 4sU enrichment of transcripts in the newly transcribed RNA fraction relative to the total RNA fraction:
    (i) Determine normalization factors K_total_ and K_newly–transcribed_ that account for the relative fraction of total volume used for reverse transcription:
    – K_total_ = V_totalRNA_/V_qPCRtotalRNA_;
    – K_newly–transcribed_ = V_newly transcribedRNA_/ V_qPCR newly transcribed RNA;_
    where
    – V_total_ = Volume of total RNA;
    – V_newly–transcribed_ = Volume of newly transcribed RNA;
    – V_qPCRtotalRNA_ = Volume of total RNA reverse transcribed;
    – V_qPCR newly transcribed RNA_ = Volume of newly transcribed RNA used for reverse transcription.
  (ii) Convert Cq/Ct to normalized expression as following: Exp_total_ = 2^–Cq total^^∗^K_total_ and Exp_newly transcribed_ = 2^–Cq^ ^newly transcribed^^∗^K_newly trascribed_
  (iii) For each transcript, calculate the ratio of normalized 4sU relative enrichment as following: 4sU_rel.–enr._ = Exp_newly transcribed_/Exp_total_.
  NOTE_25_: Gene specific 4sU_rel.–enr_ are inversely correlated with transcript half-life.

## Results

**FIGURE 2:**
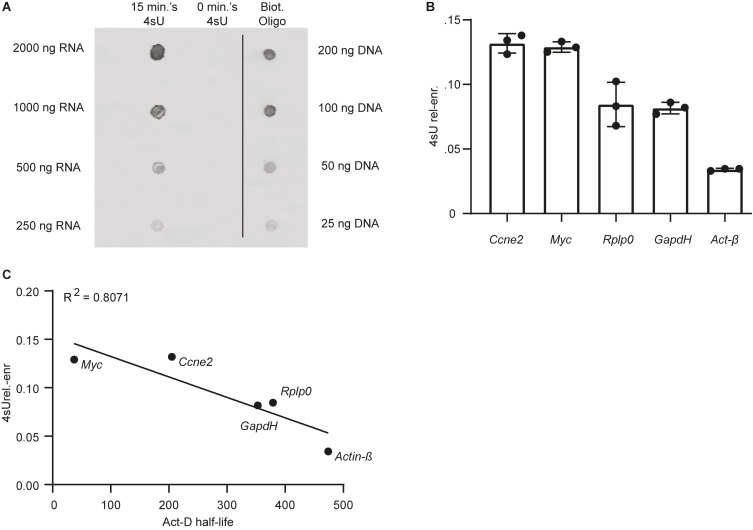
Labeling mESCs with 200 μM of 4sU for 15 min is sufficient for significant incorporation of the modified ribonucleoside in newly transcribed RNA. **(A)** Dot-Blot of RNA extracted from cells labeled with 4sU (15 min 4sU), untreated control (0 min. 4sU) and a biotinylated oligo (Biot. Oligo). 2 μl of RNA (15 min 4sU, 0 min 4sU) and biotinylated oligo (Biot. Oligo) were blotted. The respective concentrations for the blotted samples are indicated on the left and right side of the blot for the RNA samples and Biot. Oligo respectively. **(B)** 4sU relative enrichment (4sU_rel–enr_, Y-axis) of two relatively unstable transcripts (*Ccne2* and *Myc*) and three relatively stable transcripts (*Rplp0*, *GapdH*, and *Act-*β). **(C)** 4sU_rel.–enr_ (Y-axis) estimated for five genes (gene names indicated next to data point), represented as a function of half-lifes in mESC estimated using transcriptional inhibition (Sharova et al., 2009) (Act-D half-life, X-axis). The Pearson R^2^ is indicated on the top left-hand side of the plot.

We performed a Dot-Blot, as described in section C, to qualitatively assess 4sU incorporation in mESCs following labeling with 200 μM 4sU for 15 min. As expected, no clear signal was detected in the untreated mESC control. In contrast, RNA extracted from cells labeled with 4sU showed significant enrichment in biotinylated residues, as probed using streptavidin horseradish-peroxidase (Figure 2A). Following streptavidin-based separation of 4sU labeled RNA, we obtain ∼1.5% relative to input biotinylated RNA (Mass_newly–transcribed RNA_(ng)/Mass_biotinylated RNA_(ng)^∗^100) in range with what was previously reported (Marzi et al., 2016). We thus conclude that 15 min of incubation with 200 μM 4sU is sufficient to label a sizable RNA fraction in mESCs.

Subsequently we measured the relative 4sU enrichment (4sU_rel.–enr_) (Figure 2B), which correlates with transcript stability (Rabani et al., 2011; Freimer et al., 2018), for a subset of genes with different reported stabilities. As previously reported (Marzi et al., 2016), following normalization, we find that 4sU_rel.–enr_ is inversely correlated (*R*^2^ = 0.80) (Figure 2C) with previously published stabilities obtained using transcriptional inhibition in mESC (Sharova et al., 2009).

**FIGURE 3:**
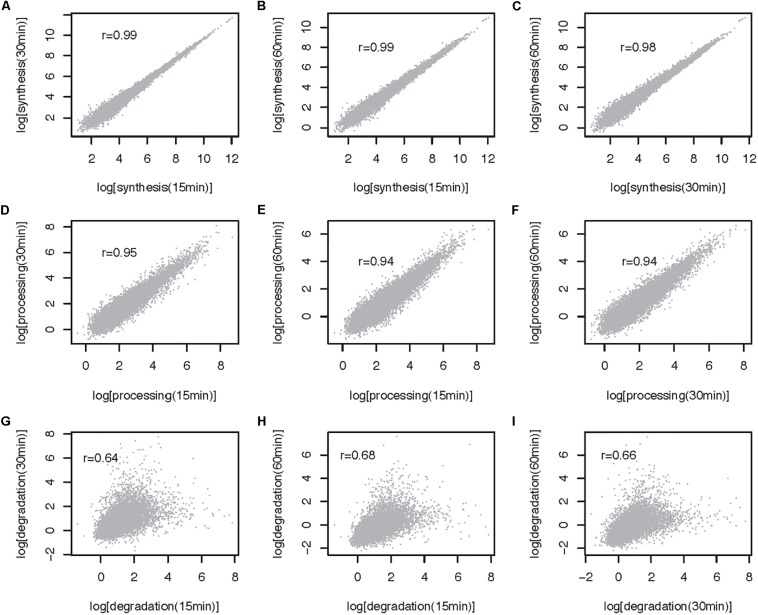
Comparison of RNA metabolic rates obtained after 15, 30, and 60 min of 4sU labeling for multiexonic mESC expressed transcripts (32 641 transcripts). **(A–C)** All against all comparison of synthesis rates (log, min^– 1^). **(D–F)** All against all comparison of processing rates (log, min^– 1^). **(G–I)** All against all comparison of degradation rates (log, min^– 1^). Each point represents one transcript. Pearson correlation (r) for each comparison is noted on the top left-hand side of the relevant panel.

**FIGURE 4:**
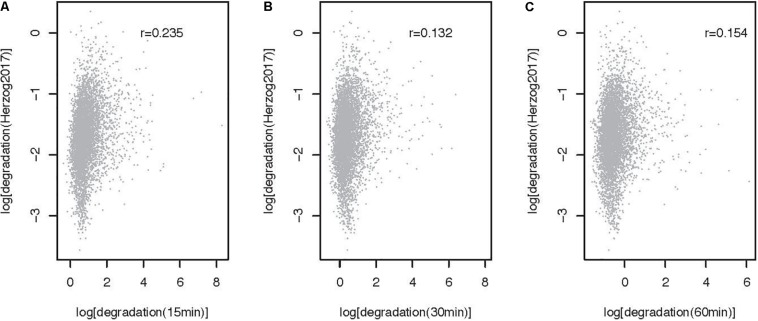
Comparison of degradation rates obtained after 15, 30, and 60 min of 4sU labeling with rates obtained using SLAM-seq (5353 transcripts). Comparison of SLAM-seq based degradation rate (Herzog et al., 2017, log, cpm/h) and rates obtained after **(A)** 15, **(B)** 30, and **(C)** 60 min of labeling with 4sU. Each point represents one transcript. Pearson correlation r for each comparison is noted on the top right-hand side of the relevant panel.

The method described here can be used to estimate RNA metabolic rates at the transcript and transcriptome wide level. To illustrate the use of the approach genome wide and to gain insights into the impact of 4sU pulse duration on rate estimates, we extracted and sequenced RNA from mESCs pulsed for 15, 30, and 60 min and estimated rates using INSPecT [(de Pretis et al., 2015), see Supplementary Note for a description of the methods]. Synthesis and processing rates are minimally impacted by 4sU pulse length, as highlighted by the high correlation we obtain between estimates obtained from the different experiments (Pearson correlation, 0.99 < *r* < 0.94; Figures 3A–F). In contrast, degradation rates estimated for cells treated with 4sU for different durations are significantly, yet less well-correlated that are the other two RNA metabolic rates (Pearson Correlation, 0.64 < *r* < 0.68, Figures 3G–I). This is expected because maximum sensitivity in decay rate estimates, for pulse-only experiments, is achieved using labeling times similar to the transcript half-life (Russo et al., 2017). Given that higher correlation is obtained between rates estimated for the shortest pulse duration (Pearson *r* = 0.235, Figure 4) and pulse-chase degradation rates estimated in mESCs using SLAM-seq (Herzog et al., 2017), we conclude that shorter pulse durations provide more accurate genome wide estimates of transcript half-lives in mESCs. The significant, yet relatively low, correlation obtained by this and published data may in part result from the use of different experimental approaches and the relatively simple assumptions, which may not faithfully recapitulate the kinetics of RNA metabolism, used by different algorithms (Duffy et al., 2019).

Furthermore, analysis of the expression of a subset of pluripotency and differentiation markers highlights that longer pulse durations lead to more pronounced differences in these markers’ expression (Supplementary Note Figures 1B,C). The small, yet significant, decrease we specifically observe in *Nanog* expression after 120 min of pulse with 4sU further underlines the advantages of using short 4sU pulse durations in mESC.

## Discussion

Gene expression is controlled by the rates at which genes are transcribed, processed, and degraded. Perturbations to any of these processes can result in changes in gene product abundance. Methods that capture the dynamic processes that control gene expression are therefore paramount to understand gene regulation. Approaches based on metabolic labeling of RNA using modified ribonuleosides, such as 4sU, bypass many of the limitations of common transcription inhibition-based approaches and are now the gold-standard in the field. However, the implementation of 4sU metabolic labeling in different cells has also brought to light some of its pitfalls. For example, differences in doubling times between different cell types, result in variation in 4sU incorporation and impact the methods’ sensitivity (Russo et al., 2017; Duffy et al., 2019). As a consequence, labeling duration and 4sU concentration must be optimized for different cell types (Dolken et al., 2008; Duan et al., 2013; Borowski and Szczesny, 2014).

Until recently, isolation of 4sU-incorporating newly synthetized RNA from preexisting RNA has been achieved through biochemical enrichment (Figure 1). This enrichment step adds experimental complexity and technical variability between replicates, which in turn decreases the accuracy of the rate estimates (Garibaldi et al., 2017; Duffy et al., 2019). The recent development of chemical-based nucleotide conversion methods, such as SLAM-seq, bypass streptavidin-dependent enrichment and provide promising alternatives to the classical approaches (Herzog et al., 2017). These methods are less labor intensive and are more technically robust. The absence of an enrichment step also ensures maintenance of the cellular ratio of newly transcribed and preexisting RNA, which in turn minimizes the requirement for normalization and simplifies computational rate inference (Uvarovskii et al., 2019).

Since its development, SLAM-Seq has been used to infer metabolic RNA rates in a variety of cellular contexts (for example, Herzog et al., 2017; Matsushima et al., 2018; Muhar et al., 2018), but still relatively little is known about how enrichment-based and chemical conversion-based methods compare. One exception, is a recent report that suggests that streptavidin-based enrichment may be preferred when using short labeling times, which is generally advised for accurate rate inference of very short or long-lived transcripts (Uvarovskii et al., 2019). Since short 4sU labeling duration results in relatively fewer labeled transcripts, in the absence of an enrichment-step, most sequencing reads will map to unlabeled preexisting RNA and will be biased towards highly expressed transcripts with slow transcription rates (Uvarovskii et al., 2019).

Because isolation of newly transcribed and preexisting RNA fractions in chemical-based nucleotide conversion methods relies on sequencing-based identification of converted sites, this method can seldomly be used to investigate the metabolic RNA rates of individual genes. This reliance on RNA sequencing, also limits the possibility of implementing quality controls prior to transcriptome-wide expression analysis. In contrast, streptavidin-dependent selection approach can be easily quality controlled, and allow testing of rates for individual transcripts as well as transcriptome wide.

The protocol described here provides guidelines for establishment of 4sU metabolic labeling in mESC, and can be adapted to other cell types and experimental designs, underlining the versatility of the technique.

## Data Availability Statement

The raw sequencing data is available on the NCBI Gene Expression Omnibus (GEO) under accession number GSE111951.

## Author Contributions

AM and AB designed the study, wrote the manuscript, read and agreed on the last version of this manuscript. AB performed the experiments.

## Conflict of Interest

The authors declare that the research was conducted in the absence of any commercial or financial relationships that could be construed as a potential conflict of interest.
