# Supplementary material for: A Protocol for Transcriptome-Wide Inference of RNA Metabolic Rates in Mouse Embryonic Stem Cells
Source: Front Cell Dev Biol. 2020 Feb 27;8:97. doi: 10.3389/fcell.2020.00097 (PMC7056730; doi:10.3389/fcell.2020.00097)
Supplement: Supplementary file 1 [file Data_Sheet_1.pdf]

Supplementary Note

Impact of 4sU labelling to mESC viability and cell state

Materials and Methods

Assessing the impact of 4sU exposure on cell viability.

Eighteen to twenty-four hours before 4sU labelling step, we seeded two gelatin-coated (.2%) 10 cm plates containing 12 ml of mESC growth medium with 4 million cells. Cells were incubated overnight at 37^oC in a humidified incubator with 5% CO2 and then labelled with 4sU, as described in Section B of the protocol. After 2 hours incubation at 37^oC, we removed medium from 4sU treated and untreated plates and washed cells with 4 ml of PBS. Plates where incubated with 2 ml of trypsin, at 37ºC for 5 minutes, and trypsin was neutralized by adding 8 ml of growth medium. The cells in 4sU treated and untreated plates were transferred to two 15 ml Falcon tubes and centrifuged for 3 minutes at 180 g. After removing the supernatant, cells were resuspended in 2 ml of growth medium and counted. For each sample, we resuspended 1 million cells in 1 ml of PBS in 1.5 ml Eppendorf tubes, added 1 µl of LIVE/DEAD green reagent (Thermo Fisher L34969), and mixed gently by pipetting. Cells were incubated at room temperature, in the dark for 30 minutes. After incubation, cells were centrifuged for 3 minutes at 180 g, and resuspended in 1.2 ml of PBS 3% (v/v) FBS and 1mM EDTA. Subsequently, each sample was separated into three technical replicates of 400 µl each.

Cells were analyzed by Flow Cytometry using a 488 nm laser as per manufacturer's instructions.

Assessing the impact of 4sU exposure on the expression levels of pluripotency and differentiation markers.

Eighteen to twenty-four hours before 4sU labelling step, we seeded two wells in a gelatin-coated (0.2%) 6-well plate containing 3 ml of mESC growth medium with 300,000 cells. Cells were allowed to grow overnight at 37^oC in a humidified incubator with 5% CO2 and labelled with 4sU, as described in Section B of the protocol for 15 minutes and 120 minutes. After incubation at 37^oC, we removed medium from 4sU

treated and untreated wells and washed cells with 1 ml of PBS. We added 350 µl Qiagen RLT directly to the wells and proceeded with cell lysis, RNA extraction and on column DNAse treatment using Qiagen Qiashredder and RNeasy Mini Kit as per manufacturer's instructions. This experiment was repeated 3 times.

We reverse transcribed 1 µg of RNA from 4sU-treated or untreated cells using Qiagen Quantitect Reverse Transcription Kit as per manufacturer's instruction (final volume 20 µl). We diluted cDNA 1:4 in DEPC-H_2O. For quantification of pluripotency and differentiation gene expression, we assembled qPCR reactions by adding 2 µl or 4 µl diluted cDNA respectively, 5 µl FastStart DNA Essential Green Master (Roche, 06402712001) and 0.5 µM of gene specific primers (primer sequences in Supplementary Table1). DEPC-H_2O was added to a final volume of 10 µl and reaction was mixed thoroughly by vortexing. qPCR reactions were performed in a Roche Lightcycler 96®, according to manufacturer's instructions, and relative gene expression levels were determined using the Roche Lightcycler 96® Software V1.1.0.1320.

Results

To assess the impact of 4sU labelling on mESC viability, we compared the number of live mESCs cells labelled with 4sU (final concentration, 200 µM) for 2 hours and untreated controls.

We observed no substantial difference in the percentage of live cells between untreated mESCs (95.1% live cells) and mESCs labelled with 4sU (200 µM) for 2 hours (96.1% live cells, two-tailed unpaired t-test P-value=0.03, Supplementary Note Figure 1A).

We analyzed the expression levels, relative to internal controls *Act-β* and *PolII*, of 4 pluripotency (*Myc*, *Sox2*, *Nanog, Oct4*) and 3 lineage specific markers (*Nestin*, *Foxa1* and *Hand1*) for mESCs treated with 4sU for 15 or 120 minutes. We found that after 15 minutes the levels of these markers were not significantly impacted relative to untreated cells (Fold-difference between expression in 15 minute 4sU treated relative to untreated cells:0.02-0.11, two-tailed paired t-test p-value 0.0624-0.6853, Supplementary Figure Note 1B-C). The expression levels of these markers changed moderately more in cells treated for 120 minutes (Fold-difference between expression in 120 minutes 4sU treated relative to untreated cells:0.04-0.35, two-tailed paired t-test p-value 0.0473-0.7334, Supplementary Figure Note 1B-C). The small, yet significant,

decrease in *Nanog* expression following a 120 minute 200 μM 4sU pulse supports that prolonged exposure to 4sU may impact gene expression programs of highly plastic cells like mESCs.

We concluded that short labelling of mESC with 4sU at a concentration of 200 μM does not significantly impact cell viability or pluripotency and differentiation genes' expression. Short pulses are therefore advisable in order to minimize changes in gene expression patterns and cell state. The present data does not exclude the potential impact of longer 4sU pulses on mESC cell state and proliferation. When implementing labelling experiments that require longer pulse durations or higher 4sU concentrations we suggest potential changes in cell cycle and stemness markers should be assessed.

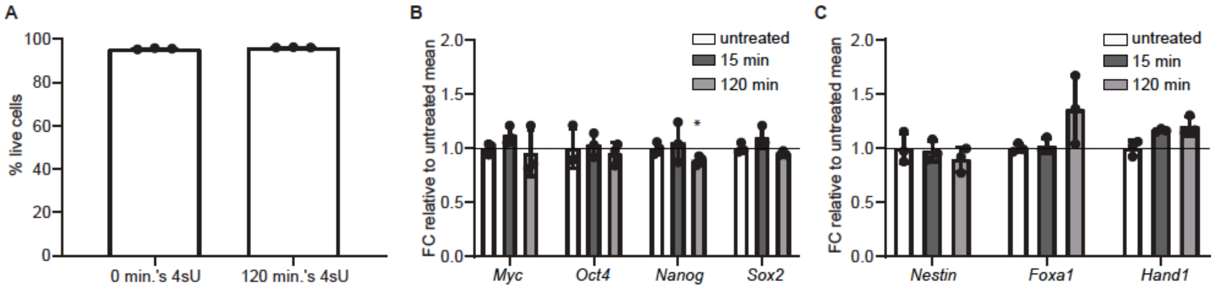

Supplementary Note Figure1: 4sU treatment does not significantly impact cell viability or cell state. A) Percentage (%) of live cells, estimated as the fraction of cells with lower fluorescent signal following staining with LIVE/DEAD GREEN in untreated mESCs or cells labelled with 4sU (200 μM) for 2 hours. B) Fold change in relative expression of pluripotency markers (*Myc*, *Sox2*, *Nanog* and *Oct4*) in cells treated with 4sU for 15 minutes and 120 minutes (15 min and 120 min respectively) relative to untreated cells. Gene expression levels were normalized by the levels of internal controls *Act-β* and *Pol II*. Dashed line represents 4sU treated/ untreated value of 1.0. C) Fold change in relative expression of differentiation markers (*Nestin*, *Foxa1*, *Hand1*) in cells treated with 4sU for 15 minutes and 120 minutes (15 min and 120 min, respectively) relative to untreated cells. Gene expression levels were normalized by the levels of internal controls *Act-β* and *Pol II*. Dashed line represents 4sU treated/ untreated value of 1.0. (Statistics: two-tailed paired p-value<0.05-*, p-value>0.05 are not represented)

Genome wide inference of RNA metabolic rates

Materials and Methods

Mouse DTCM23/49 XY embryonic stem cell lines (mESCs) were cultured at 37°C with 5% CO_2 in Knockout Dulbecco's Modified Eagle Medium (D-MEM, Thermo Fisher, #10829-018) supplemented with 15% fetal bovine serum (FBS, Thermo Fisher, #16000-044), 1% antibiotic penicillin/streptomycin (Thermo Fisher, 15070063), 0.01% recombinant mouse LIF protein (Merck, #ESG1107) and 0.06 mM 2-Mercaptoethanol (Thermo Fisher, #31350-010), on 0.1% gelatin-coated cell culture dishes. When confluent, culture was divided in two and passaged 8 times. Five million mESCs of two biological replicates were seeded and allowed to grow to 70-80% confluency (approximately 1 day). RNA was labeled by adding 4sU to the growth medium (final concentration of 200 µM) and incubating cells at 37 ºC for 15, 30 or 60 minutes. Total and nascent RNA was isolated as described in the manuscript methods section.

Total RNA libraries were prepared from 10 ng of DNase-treated total and newly transcribed RNA using Ovation® RNA-Seq and sequenced on Illumina HiSeq 2500 (average of fifty million reads per library).

Hundred nucleotides long single-end stranded reads were first mapped to mouse ribosomal RNA sequences with STAR v2.5.0 (Dobin et al., 2013). Reads that did not map to ribosomal RNA were then aligned to intronic and exonic sequences using STAR and quantified using RSEM (Li and Dewey, 2011). Rates of synthesis, processing and degradation were independently inferred using biological duplicates at each labeling point using the INSPEcT Bioconductor package v1.8.0 (de Pretis et al., 2015). The raw sequencing data is available on the NCBI Gene Expression Omnibus (GEO) under accession number GSE111951.
